# Circulating Thrombomodulin: Release Mechanisms, Measurements, and Levels in Diseases and Medical Procedures

**DOI:** 10.1055/a-1801-2055

**Published:** 2022-07-11

**Authors:** Mallorie Boron, Tiffany Hauzer-Martin, Joseph Keil, Xue-Long Sun

**Affiliations:** 1 Department of Chemistry and Chemical and Biomedical Engineering and Center for Gene Regulation in Health and Disease (GRHD), Cleveland State University, Cleveland, Ohio, United States

**Keywords:** circulating thrombomodulin, COVID-19, endothelial cell, microvesicle, sepsis, soluble thrombomodulin, thrombomodulin, vascular damage

## Abstract

Thrombomodulin (TM) is a type-I transmembrane protein that is mainly expressed on endothelial cells and plays important roles in many biological processes. Circulating TM of different forms are also present in biofluids, such as blood and urine. Soluble TM (sTM), comprised of several domains of TM, is the major circulating TM which is generated by either enzymatic or chemical cleavage of the intact protein under different conditions. Under normal conditions, sTM is present in low concentrations (<10 ng/mL) in the blood but is elevated in several pathological conditions associated with endothelial dysfunction such as cardiovascular, inflammatory, infection, and metabolic diseases. Therefore, sTM level has been examined for monitoring disease development, such as disseminated intravascular coagulation (DIC), sepsis and multiple organ dysfunction syndrome in patients with novel coronavirus disease 2019 (COVID-19) recently. In addition, microvesicles (MVs) that contain membrane TM (MV-TM) have been found to be released from activated cells which also contribute to levels of circulating TM in certain diseases. Several release mechanisms of sTM and MV-TM have been reported, including enzymatic, chemical, and TM mutation mechanisms. Measurements of sTM and MV-TM have been developed and explored as biomarkers in many diseases. In this review, we summarize all these advances in three categories as follows: (1) release mechanisms of circulating TM, (2) methods for measuring circulating TM in biological samples, and (3) correlation of circulating TM with diseases. Altogether, it provides a whole picture of recent advances on circulating TM in health and disease.

## Introduction

### Thrombomodulin in General

**Fig. 1:**
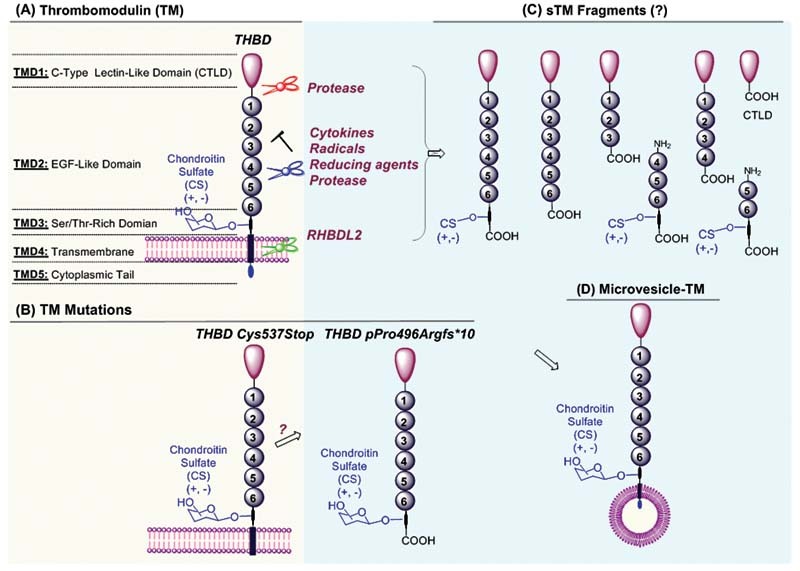
Schematic presentation of structural domains of membrane thrombomodulin (TM) ( **A** ) and TM mutations ( **B** ), its release mechanisms of predicted sTMs with corresponding domains (C), and microvesicle-TM ( **C** ). CS, chondroitin sulfate; CTLD, C-type lectin-like domain; EGF, epidermal growth factor; RHBDL2, the intramembrane protease rhomboid-like-2; Ser, serine; sTM, soluble thrombomodulin; Thr, threonine.

Thrombomodulin (TM) is a type-I transmembrane glycoprotein that was first discovered by Esmon and Owen in 1981 on endothelial cells as a cofactor for thrombin-catalyzed activation of protein C. 1 This protein is encoded by an intronless gene ( *THBD* ) located on the chromosome 20p12-cen. 2 Since its original identification, TM has also been found on a large variety of cells including macrophages, monocytes, platelets, neutrophils, and mesothelial cells. 3 4 5 Endothelial TM is a made up of 557 amino acids with a molecular weight of approximately 74 kDa. 6 There are five total domains that make up the total structure of mature TM ( Fig. 1 ). Starting from the *N* -terminus, these domains are the lectin-like domain (TMD1), epidermal growth factor (EGF)-like domain (TMD2), serine/threonine-rich domain (TMD3), transmembrane domain (TMD4), and a cytosolic tail (TMD5; Fig, 1A ). 6 TM has multiple biological functions which are attributed to its different domains. 7 The lectin-like domain of TM is similar in structure to C-type lectins but lacks a calcium-binding site (named as C-type lectin domain [CTLD]). This domain is involved in inflammation, tumor growth, and cell adhesion. It exerts anti-inflammation actions by binding proinflammatory stimuli before they can reach their target. These include lipopolysaccharide (LPS) and high-mobility group box 1 protein. 8 9 For its role in cell adhesion, the lectin-like domain can bind to fibronectin of the extracellular matrix. 10 The TMD2 domain of TM contains six EGF-like repeats and is the site for TM's anticoagulation and fibrinolysis functions. These functions are allowed by TM's ability to activate protein C for anticoagulation, anti-inflammation, and thrombin activatable fibrinolytic inhibitor (TAFI) activation for fibrinolysis. 11 Both of these processes require thrombin, which requires EGF56 for binding. 12 13 It has been elucidated that the minimum structure for protein C activation is EGF456, while TAFI activation requires EGF3456. 14 15 16 In addition to the coagulation function, the TMD2 domain has mitogenic activity, although the exact repeats needed for this activity is unknown. 17 18 Next, the TMD3 domain is a serine/threonine-rich domain which contains attachment sites for chondroitin sulfate (CS). 19 20 There are two kinds of membrane TM, one with and one without CS. 21 The CS moiety of TM is important for the enhancement of protein C activation by the thrombin–TM complex. 22 23 The TMD4 domain anchors TM to the cell membrane and classifies TM as a type-I membrane protein. 24 The TMD5 domain is a small cytoplasmic tail region that plays a role in TM's ability to multimerize. 25 26

### Circulating Thrombomodulin in General

In addition to expression as a membrane protein on the cell surface, fragments of TM are also found circulating in the blood, 27 urine, 28 and other biofluids. 29 These fragments of TM lack the transmembrane domain and are known as soluble TM (sTM). They are derived from membrane TM by cleavage via either proteolysis or chemical and physical stress ( Fig. 1B ). The sTM consists of fragments of different molecular weights whose presence can vary by disease. 29 30 Different levels of sTM are found in many diseases. 31 32 33 In addition, endothelial cells can release microvesicles (MVs) containing membrane TM (microvesicle-TM) which also contribute to circulating TM levels ( Fig. 1C ). 34 Understanding of TM release mechanism is critical to comprehend its significance and role in disease development.

Measurement of the levels of circulating TM has great potential as a biomarker for diagnosis and tracking of different diseases. In this review, we summarize all these advances in three categories: (1) release mechanisms of circulating TM, (2) methods for measuring circulating TM in biological samples, and (3) correlation of circulating TM with diseases.

### Release (Shedding) of Thrombomodulin

In healthy humans, the levels of sTM are low (<10 ng/mL), 35 while high sTM levels are common in patients suffering from various diseases. The mechanism responsible for sTM release is complex and several mechanisms have been proposed and confirmed. Primarily, TM is shed from the cell by enzymatic and/or chemical cleavage. It is known that endothelial TM serves as a cellular substrate for proteolytic cleavage, frequently leading to its shedding as various forms of sTM. Also, chemical cleavage of membrane-bound protein can generate sTM. Increased plasma sTM level has been accepted as a sensible marker for endothelial damage. 36 In particular, the consistent elevation of sTM levels during pathologies is now widely regarded as an important circulatory biomarker for endothelial dysfunction and vascular risk assessment. 37 38 39 It has been shown that sTM levels correlate with disseminated intravascular coagulation (DIC), stroke, multiple organ failure and mortality. 40 41 42 43 In addition, TM mutation that causes a synthesis of TM with the decreased size of the transmembrane domain can also contribute to the high levels of sTM in plasma. 44 A new autosomal dominant bleeding disorder characterized by very high plasma levels of sTM has been reported, in which the *THBD* c.1611C > A (p.Cys537X) mutation in a heterozygous state was identified. 45 46 The mutated TM lacks the last three amino acids of the transmembrane domain and the cytoplasmic tail and is associated with an increase in sTM in the plasma. On the other hand, activated endothelium can release microvesicles (and exosomes) containing membrane TM (microvesicle-TM). 47 Overall, the mechanism responsible for TM shedding is complex and is not completely understood. Both the extracellular stimuli and a defect of synthesis of truncated TM contribute to the high levels of sTM in plasma. Understanding the mechanisms for TM shedding could help better understand underlying mechanisms of many diseases. This section summarizes various mechanisms for TM shedding known so far.

#### Proteolytic Release of Soluble Thrombomodulin

Previous research focused primarily on the proteolytic release of soluble TM from cells, which exists in biological fluids such as plasma, urine, and synovial fluid. 25 26 27 Proteolytic release of TM is a process, by which TM is cleaved from the cell surface after being exposed to specific proteases. 47 It is known that sTM is generated by proteolytic cleavage by proteases released during disorders associated with vascular damage, which include infection, sepsis, and inflammation. 48 It is known that neutrophil-derived proteases, 27 49 rhomboids, 50 metalloproteinases, 51 52 and possibly also cytokines 53 54 55 can cleave TM from the surface of endothelial cells. An earlier study demonstrated that primed activated neutrophils are potent modulators of endothelial TM using an endothelial tissue culture system. 49 In particular, neutrophil derived elastase and cathepsin G caused rapid dose-related reduction of TM activity on endothelial cell surface. The full-length TM extracellular domain (ECD) has also been shown to be cleaved from the endothelial cell surface after incubation with the neutrophil protease elastase, cathepsin G, and proteinase 3. 27 In addition, TM CTLD can also be cleaved from the cell surface by matrix metalloproteinases (MMPs). 55 A Recent research confirmed that TM is a specific substrate of a transmembrane serine protease known as rhomboid-like-2 (RHBDL2). 56 RHBDL2 cleaves TM at a site proximal to the transmembrane domain, resulting in release of the ECD. 50 Furthermore, several inflammatory processes are associated with a moderate but statistically significant increase of sTM levels in plasma due to the proteolysis of TM by different leukocyte-derived proteases (elastase and cathepsin). 57 In the case of cytokine-induced release of TM, a metalloproteolytic cleavage mechanism was proposed in which cytokine induces metalloproteinase expression. 52 Exposure of cytomix (tumor necrosis factor [TNF]-α, interleukin [IL]-1β, and interferon-γ) to model alveolar epithelium A549 cells, primary human small airway epithelial cells, and primary human alveolar epithelial type-II cells induced shedding of TM. The shedding of TM was blocked by the hydroxamic-based metalloproteinase inhibitors TAPI and GM6001, suggesting that shedding of TM is mediated by a metalloproteinase. 52 However, no specific metalloproteinase was identified for the cytokine-induced metalloproteolytic cleavage of TM yet. Overall, the proteolytic release of TM depends on specific enzymes which afford different fragments of TM. Reported enzymatic release mechanisms of TM are summarized in Table 1 . The physiological and pathological relevance of sTM release with different fragments requires further investigation.

**Table 1:** Proteolytic and nonproteolytic release of membrane bound TM

Release mechanism		Source	sTM (MW)	References
Enzymatic	Metalloproteinases	HUVEC	60 kDa	51
	Neutrophil derived proteases	HUVEC	56 kDa NA	27 49
	Rhomboids	Keratinocytes	90 kDa	50 56
Cytokine	TNF-α	HUVEC	NA	54
	Cytomix (TNF-α, IL-1β, and Interferon-γ)	Lung epithelial cells	NA	52 53
Chemical	Glutathione	HUVEC	NA	58
	Lysophosphatidic acid	HUVEC	63 kDa	58
	Oxygen radicals	HUVEC	56 kDa NA	27 49
	H _2_ O _2_	HUVEC	NA	57
Physical	Cyclic strain	HUVEC	NA	62
Microvesicles		Monocyte (LPS)	NA	67
		Blood (Baboon after severe heatstroke)	NA	68
		Blood (SIRS patients)	NA	69
		HUVEC (cyclic strain)	NA	62
TM mutation		Blood and COS-1 cells	NA	44 45 46 76

Abbreviations: IL, interleukin; LPS, lipopolysaccharide; MW, molecular weight; NA, not available; sTM, soluble thrombomodulin; TNF, tumor necrosis factor; HAEC, human aortic endothelial cell; SIRS, systemic inflammatory response syndrome

#### Chemical Release of Soluble Thrombomodulin

sTM can also be generated by chemical cleavage of the membrane bound TM ( Table 1 ). It has been shown that reducing agents such as glutathione, dihydrolipoic acid, and acetylcysteine (nonprotein thiols) can effectively stimulate release of sTM into the cell culture medium of human aortic endothelial cells (HAECs). 58 The use of reducing agents to release sTM will inactivate TM. In addition, oxygen radicals are known to rapidly induce direct toxic effects on endothelial cells (cytolysis) in vitro at high concentrations. This endothelial cell cytotoxicity is closely related to an increase in sTM levels in the culture supernatant after treatment of endothelial cells with oxygen radicals. 27 It is well known that oxygen and other free radicals oxidize Met388 in TM, thereby reducing the activation of protein C by 90% 59 while not affecting activation of TAFI. 16 Therefore, sTM released by oxygen radicals may have its Met388 oxidized and thus have less protein C activation activity as well. Furthermore, lysophosphatidic acid (LPA), a bioactive lipid mediator, is present during endothelial damage or injury. Treatment with LPA leads to shedding of the lectin-like domain of TM in HUVECs. 55 Currently, there is no report on chemical release of sTM with different fragments in vivo and its pathological relevance, which requires further investigation.

#### Physical Release of Soluble Thrombomodulin

Proteolytic and chemical release are the main contributors of sTM in the blood. However, physical force from the blood flow also causes sTM release from endothelial cells. It is known that blood flow–associated hemodynamic forces, such as cyclic strain (stretch) and shear stress, affect endothelial-dependent regulation of vessel homeostasis. 60 The effect of hemodynamic forces on endothelial TM expression has been investigated. 61 It was found that physiologic hemodynamic forces (cyclic strain) causes TM release from endothelial cells. The effects of equibiaxial cyclic strain and laminar shear stress on TM expression and release was examined with HAECs in vitro. 62 As a result, physiologic cyclic strain could cause the release of sTM in a time-, dose-, and frequency-dependent manner. There was no proteolytic release of sTM observed as inhibition of either MMPs (GM6001) or rhomboids (3,4-dichloroisocoumarin) showed no effect on strain-induced sTM release. This study indicates the importance of physical force on TM expression and release in vivo, especially in pathological and surgical operation procedures which requires further study.

#### Release of Microvesicle-Thrombomodulin

MVs, a population of extracellular vesicles ranging in size from 0.1 to 1 μm, are released from the surface of cells by the process of outward membrane budding through a loss of calcium-dependent membrane phospholipid asymmetry and cytoskeletal rearrangement. 63 64 MVs are released from the cell surface in response to cellular activation or apoptosis and are found in blood circulation at low levels during normal physiologic conditions, but at elevated levels in a variety of diseases. 65 66 In body fluids, they constitute reliable hallmarks of cell damage. Early work by Satta and coworkers demonstrated that LPS treatment increases TM activity on monocyte-derived MVs by up to 80%. 67 Coelevated TM and MVs levels in serum have also been observed during heat stroke in baboons. 68 69 A later work by Duchemin et al pointed to an influence of circulating MVs on the “TM resistance” of patients suffering from myeloproliferative neoplasm. 70 MV-TM release from activated endothelium via endothelial MVs has been observed. 71 It was reported that endothelial injury releases MV-TM and MVs presenting other cell-specific surface antigens in the pathogenesis of sepsis. 34 They found that amount of the MV-TM was increased significantly in severe sepsis patients versus those in healthy controls, suggesting that it may play a role in the progression of sepsis-induced DIC. In another study, significantly elevated levels of MV-TM was isolated from HUVECs following physiologic cyclic strain. 64 Recently, MV-TM has been examined as potential biomarkers in sepsis, 72 cirrhosis, 73 and hepatocellular carcinoma (HCC). 74 The biological activity of MV-TM is still unclear but is speculated to affect vascular homeostasis. Therefore, a clearer understanding of how MV-TM is regulated within the vascular endothelium by physiological and pathological factors is of significant interest.

#### Thrombomodulin Mutation and Shedding

TM mutations impair its function and are related to diseases development. 75 TM mutations are also associated with high levels of plasma sTM. 44 45 46 76 To date, two TM mutations have been demonstrated to cause high levels of possessed plasma sTM. *THBD* Cys537Stop is the first identified TM mutation that arises from a premature stop codon at Cys537, resulting in truncation of TM within the transmembrane domain 45 ( Fig. 1B ). It was found that each affected individual possessed plasma sTM levels > 100-fold higher than that normally observed. The mechanism by which the TM Cys537Stop is more readily released from the cell surface is still not fully understood, but may be associated with decreased membrane stability or increased susceptibility to proteolysis from membrane or plasma proteases. 45 46 A recent study by Westbury et al identified a novel TM mutation ( *THBD* pPro496Argfs*10) that results in a stop gain that causes synthesis of a TM variant truncated at the membrane-proximal C-terminal region of the extracellular domain 76 ( Fig. 1B ). This truncated TM variant is therefore presumably no longer membrane localized and is instead secreted directly into the bloodstream, resulting in plasma sTM levels >100-fold higher than normal. These two mutations have been demonstrated to cause TM-associated coagulopathy that was first described in a family exhibiting abnormal bleeding that could not be attributed to known coagulation disorders by routine laboratory analyses. 77

### Measurement of Circulating Thrombomodulin and its Activity in Biological Samples

Circulating TM exists either as sTM cleaved from membrane TM or MV-TM that is membrane TM released from cell membrane. Therefore, different methods are used to quantify their concentrations in biological samples. The level of sTM has been examined as a parameter of disease severity and progression. However, determination of whether sTM levels change between healthy and patients may have mixed results in certain diseases. Alternatively, TM indexes, which compares sTM and albumin levels in serum or other biofluid, have been used. 78 79 In addition, sTM concentration and TM activity are measured in biological samples and are used together for various diseases. In this section, the methods for quantifying sTM and MV-TM concentration and their activity are discussed in detail.

#### Methods for Quantification of Soluble Thrombomodulin in Biological Samples

The most common techniques used to measure the protein levels of sTM are enzyme immunoassays (EIA) and enzyme-linked immunosorbent assays (ELISA). In addition, western blot and high-performance liquid chromatography (HPLC) methods are also used to quantify sTM concentration in biological samples. This section summarizes common methods for detecting and measuring sTM concentrations in biological samples ( Table 2 ).

**Table 2:** Methods for measuring sTM in biological samples and diseases

Method	Sample	Disease(s)	Reference(s)
EIA	Plasma	Atherosclerosis, diabetes, DIC, sepsis	40 111 116
	Serum	Atherosclerosis, diabetes	127 129
	Urine	Diabetes	127
	Cerebral Spinal Fluid	Multiple sclerosis	78
ELISA	Plasma	ARDS, CAP, CHD, COVID-19, diabetes, HUS, hypertension, preeclampsia, lupus, multiple sclerosis, SARS, sepsis, stroke, TTP	43 53 91 93 99 100 104 105 119 120 121 128 148 149 150 159 160 161 192
	Pulmonary edema fluid	ARDS	53
	Serum	Lupus, multiple sclerosis, sepsis	78 95 106
HPLC-UV/Vis	Purified protein	Diabetes	30
Western Blot	Protein extract	Diabetes, transplant	193 194

Abbreviations: ARDS, acute respiratory distress syndrome; CAP, community-acquired pneumonia; CHD, coronary heart disease; COVID-19, novel coronavirus disease 2019; DIC, disseminated intravascular coagulation; EIA, enzyme immunoassays; ELISA, enzyme-linked immunosorbent assays; HPLC, high-performance liquid chromatography; HUS, hemolytic uremic syndrome; SARS, severe acute respiratory syndrome; TM, thrombomodulin; TTP, thrombotic thrombocytopenic purpura; UV/Vis, Ultraviolet–visible

##### Enzyme Immunoassays/Enzyme-Linked Immunosorbent Assays

EIA and ELISA methods are the most common ways to measure the concentration of sTM ( Supplementary Table S1 ). The detection limits can reach the pg/mL range but most often read in the low ng/mL range. Another advantage is the need for little sample preparation before analysis. In theory, only sTM should be binding to the detecting or capturing antibodies and all other molecules are washed away and thus undetected. This allows for the immunoassays to handle complex samples such as plasma and urine. So far, many ELISA kits have been developed and are commercially available now ( Supplementary Table 1 ). These ELISA kits use biotin- and horseradish peroxidase (HRP)-labeled anti-TM antibodies or biotin-labeled detection antibodies to detect the sTM in serum, plasma, cell culture supernatant, tissue, or other fluids. However, there is no information available related to the specificity of TM domains for all these ELISA kits. If the presented sTM fragments do not contain the specific domain recognized by the antibodies, they will not be detected and quantified. In addition, to the reviewers' knowledge, no manufacturers of the commercial ELISA kits provide proof that the dose response for each of the sTM fragments is identical. Also, the identity of the antibodies used in the kits is often not disclosed, not even if they are monoclonal or polyclonal. Another issue is that the different suppliers offer very different levels of information on specificity, validation, and reproducibility. Therefore, the interpretation of changes in levels of sTM always needs to be cautious since the change of sTM fragments may cause ELISA response without the change of the overall level of sTM released.

##### Western Blot

Western blot is another antibody-based assay that is used to identify the presence of sTM in samples. In general, the initial gel electrophoresis step separates proteins based on their molecular weights. Transferring the separated protein to a membrane and probing with antibodies against sTM allows for the identification of sTM subspecies. The major advantage of using western blot is the ability to determine different molecular weight species of sTM if they all contain a fragment that can be recognized by an antibody. While the ability to see the different sTM subspecies of TM is helpful, western blot is not the best for gaining quantitative information. In addition to pictorial data, western blot data are represented as a ratio of the protein of interest to a loading control. Thus, western blot offers semiquantitative and most importantly qualitative information.

##### High-Performance Liquid Chromatography

HPLC offers the benefit of obtaining both quantitative and qualitative data at once. Detection limits of HPLC analysis with a UV-Vis detector can be similar to those of EIA/ELISA and reach the low ng/mL range. 30 Size exclusion chromatography allows for separation and identification of different molecular species of sTM. Molecular weights can be determined by comparing retention times of detected subspecies to those of standard proteins of known molecular weight. This gives the advantage of identifying molecular subspecies of sTM and their concentrations in the sample. However, the main disadvantage is that the sample needs to be pure sTM protein. The sTMs must first be isolated from the more complex sample using a method such as immunoprecipitation. The reasoning behind this is that other proteins could be coeluted with the sTM subspecies and give false readings. Another disadvantage is the need for method development and column selection and availability. Developing methods for HPLC is more time consuming and complicated than those for ELISA and western blotting. Finally, HPLC analysis under nonreducing and nondenaturing conditions could not provide the sequence information of sTMs.

#### Measurement of Microvesicle-Thrombomodulin in Biological Samples

Flow cytometry is a conventional analytical technique for measuring physical and chemical characteristics of a population of cells or particles. In general, cell or particle surfaces are often labeled with fluorescent markers with defined excitation and emission wavelengths. Cells or particles are then quickly examined, and the data gathered are processed by a computer software. TM-presenting endothelial MVs were found in sepsis-induced DIC. 34 TM-presenting endothelial MVs were measured by flow cytometry. 72 73 74 Various MVs from different cells were labeled with different antibodies. Annexin V, anti-CD146 antibody, and anti-CD141 antibody (BD Biosciences) were used to stain the endothelial MVs. Specifically, Annexin V, anti-CD146 antibody, anti-CD141 antibody, and antiCD201 antibody (BD Biosciences) were used to stain the microvesicles. Endothelial MVs were defined by detecting annexin V and CD146 on the vesicle surface. TM-presenting endothelial MVs were defined by detecting annexin V, CD146, and CD141 on the particle surface. 34 It should be pointed out that there is no method developed so far to quantify the mass of TM-presenting MVs compared with freely circulating sTM, which is needed to evaluate its physiological and pathological relevance.

#### Measurement of the Activity of Circulating Thrombomodulin in Biological Samples

Membrane TM has many biological activities which depend on its different domains and expression by different types of cells. 47 A variety of sTMs are released from membrane TM. It is important to know whether the sTM fragments have intrinsic activities. In the first report of sTM, Ishii and Majerus observed that sTM is less active than cellular TM in activating protein C. 28 Later studies measured the cofactor activity of isolated sTM from plasma, and found that the protein C activity was 30 to 50% compared with that of cellular TM. 28 30 35 80 Isolated human urinary sTM also shows protein C activation in human plasma. 81 As mentioned earlier, the minimum binding domain of TM for protein C activation is EGF456, 14 15 16 therefore, measurement of protein C activation is limited to the sTM containing EGF456 only. Previous studies measured the activity of sTM in biological fluids had not been investigated systemically under physiological or pathophysiological conditions. Therefore, the physiological and pathological significance of circulating and urinary sTM is presently unclear, which deserves future deeper research.

Measurement of the level of circulating sTM by immunological assays can be used to indicate endothelial-cell damage; however, it is not enough to fully evaluate certain diseases. 82 In addition, the use of sTM levels as a marker of endothelial injury is complex in certain patients like children, since it is physiologically increased during the first years of life. 83 Schneider et al analyzed the variations of sTM activity (TMa) and sTM antigen levels (TMag) in plasma of children with autologous and allogeneic bone marrow transplantation (BMT) and evaluated the ratio of TMa/TMag, since they observed that it was independent of age in healthy children. 84 In brief, TMa levels were measured on the STA-R analyzer (Diagnostica Stago, Asnières, France) using a chromogenic assay based on the ability of sTM to activate protein C after incubating with thrombin, protein C, polybrene, and a fibrin polymerization inhibitor. The activity was monitored with an activated Protein C (APC) substrate (CBS 4246) at 405 nm. It was found that the ratio of TMa/TMag could constitute a marker for an early discrimination of children with high risk of complications during allogeneic BMT. Another study by Rousseau et al demonstrated that the measurement of plasma TMa and TMag could provide best discrimination between preeclampsia and normal pregnancy. 85 It was found that TMag and TMa levels increased in normal pregnancy but a significant increase was observed in preeclampsia which could be due to a more pronounced injury of the endothelium than in normal pregnancy. The TMag and TMa levels were also used to assess the prognosis of acute myocardial infarction. 86

### Circulating Thrombomodulin in Diseases and Medical Procedures

As mentioned above, shedding of sTM and subsequent increased levels of sTM is mainly associated with endothelial injury or damage. Therefore, many diseases show high level of sTM in serum, urine, and other biofluids. Measurement of levels of sTM in serum, urine, and other biofluids are often taken for disease diagnostic and development monitoring, as well as therapeutic monitoring. Circulating sTM levels have been measured in many diseases and medical procedures, such as infectious disease, cardiovascular disease, diabetes, hypertension, obesity, immune diseases, surgical operation, transplantation, and hemodialysis (HD). Most diseases show high levels of circulating sTM, while some diseases show lower than base levels of circulating sTM. The majority of sTM levels are measured in either serum or plasma. The levels of sTM in plasma and in serum often cannot be compared directly often. If the detection method uses an enzyme, then ethylenediaminetetraacetic acid (EDTA) might not be the best choice, since it can inhibit enzyme activity. It should also be pointed out that sTM level alone cannot be used for clinical decision. Other biomarkers are often monitored with sTM as well. This section discusses the correlation of the circulating TM and (1) diseases, (2) surgical operation and intervention, and (3) HD in detail. The sTM levels and major diseases are summarized in Table 3 .

**Table 3:** sTM and other markers in major diseases

Disease	Sample(s)	sTM Level ↑ (increase/↓ decrease)	Other markers	Reference(s)
AAA	Plasma	↑	Fibrinogen, D-dimer, CRP	86 108 195
ARDS	Edema fluid, plasma	↑	vWF, P/E-selectin	53 91 196 197
Atherosclerosis	Plasma, serum	↑	CRP, proinflammatory cytokines, fibrinogen	111 192 198
CAP	Plasma	↑	PCT, CRP, copeptin	99 199
Cardioembolic stroke	Plasma	↑	D-dimer, TAT, vWF	43 167 200
CHD	Plasma	↓	Insulin, GHS-Px, TNF-α	111 201
COVID-19	Plasma	↑	vWF, P-selectin	93 94
Diabetes	Plasma, serum, urine	↑	HbA1c, AGEs,	30 112 121 122 123 202
DIC	Plasma	↑	TAT, PIC, D-dimer	40 41 101 116 117
HUS	Plasma	↑	MMP-3, sTNFRII, sIL-6R	119 120 203
Hypertension	Plasma, serum	↑	CRP, PAI-1	119 129 130 204
Lupus	Plasma, serum	↑	Anti-dsDNA, ANAs	148 193 205
Multiple sclerosis	Cerebral spinal fluid, plasma	↑	Oligoclonal bands, antibodies (anti-MOG, anti-AQP-4)	78 79 150 206
Obesity	Plasma	↑	NA	154
Preeclampsia	Plasma	↑	sFLT-1, sEng	158 159 160 207
Sepsis	Plasma, serum	↑	CRP, PCT, proinflammatory cytokines	40 92 93 104 105
SARS	Plasma	↑	Nucleocapsid protein	100 208
TTP	Plasma	↑	Troponin I, anti-vWFCP antibody	119 209 210

Abbreviations: AAA, abdominal aortic aneurysm; ACI, acute cerebral infarction; AGEs, advanced glycation end products; ANAs, antinuclear antibodies; AQP-4, aquaporin-4; ARDS, acute respiratory distress syndrome; CAP, community-acquired pneumonia; CHD, coronary heart disease; COVID-19, novel coronavirus disease 2019; CRP, C-reactive protein; DIC, disseminated intravascular coagulation; GHS-Px, glutathione peroxidase; HbA1c, hemoglobin A1c; HUS, hemolytic uremic syndrome; MMP-3, matrix metalloprotease protein-3; MOG, myelin oligodendrocyte glycoprotein; PAI-1, plasminogen activator inhibitor-1; PCT, procalcitonin; PIC, plasmin-α2-plasmininhibitorcomplex; SARS, severe acute respiratory syndrome; sEng, soluble endoglin; sFLT-1, soluble FMS-like tyrosine kinase; sIL-6R, soluble interlukin-6 receptor; sTNFRII, soluble tumor necrosis factor receptor type II; TAT, thrombin-antithrombin complex; TNF-a, tumor necrosis factor α; TTP, thrombotic thrombocytopenic purpura; vWF, von Willebrand's factor; vWFCP, vWF cleaving protease.

#### Circulating Thrombomodulin in Diseases

##### Infection and Lung Diseases

Acute lung injury and acute respiratory distress syndrome: the pathogenesis of acute respiratory distress syndrome (ARDS) is linked to a series of inflammation reactions that lead to the accumulation of neutrophils in the lungs 87 which causes endothelial cell damage in the lungs. 88 The lungs are rich in TM and have one of the highest levels of TM among human organs. 89 90 Since the pathogenesis and clinical displays of ARDS are closely linked to endothelial cell damage, sTM has been explored as a possible biomarker for disease severity and progression. It has been shown that patients with ARDS or those who are at risk for developing the condition have increased sTM levels in plasma and pulmonary edema fluid. 53 Patients already diagnosed with ARDS with more severe complications were seen to have higher levels of sTM compared with those who had less severe manifestations. 85 Also, patients who died of complications due to ARDS had significantly higher levels of sTM compared with those who survived. 91 In addition to increased plasma levels of sTM, it has also been shown that lung sections of ARDS patients had lower expression of TM compared with healthy patients. 92 This observation agrees well with the trend of seeing increased blood levels of sTM in ARDS patients. Overall, plasma sTM level can be a useful predictor for the onset of ARDS, as it proves to be a good addition to other clinical markers and tests to help determine disease severity.

Novel coronavirus disease 2019: The novel coronavirus disease 2019 (COVID-19) is a new infectious disease caused by severe acute respiratory syndrome-coronavirus-2 (SARS-CoV-2) which has been producing devastating effects not only on human health but also on the global economy. Clinical studies showed that endothelial vascular injury plays a key pathogenetic role in the development of COVID-19-associated coagulopathy, especially among intensive care unit (ICU)-hospitalized patients. 93 Markers of endothelial cell injury could be used to identify the disease severity and mortality. Two recent findings suggest that the plasma level of sTM is highly correlated with survival among COVID-19 patients and measuring sTM levels might aid in managing patients. 93 94

A report by Goshua et al from Yale University examined blood samples of COVID-19 patients (those critically ill in an (ICU) and others receiving care but in a non-ICU unit) and disease-free volunteers. 93 95 Specifically, they compared biomarkers of endothelial cell and platelet activation, including sTM, von Willebrand's factor (vWF) antigen, soluble P-selectin, and soluble CD40 ligand, as well as coagulation factors, endogenous anticoagulants, and fibrinolytic enzymes. They found that markers of endothelial cell and platelet activation were significantly elevated in patients, ICU patients versus non-ICU patients. In particular, ICU patients with high sTM levels were discharged from hospital to a significantly lesser degree than those with lower sTM levels, while in a total patient cohort and ICU-only cohort, high sTM levels were consistently associated with decreased survival probability. From the pathophysiological perspective, elevated sTM concentrations likely reflect direct endothelial cell damage, therefore the concentration of sTM in the blood might be the surrogate for the degree of endothelial injury in COVID-19. Moreover, as a surrogate marker of endothelial injury, sTM also seems to provide prognostic information in this population. In another study, Jin et al analyzed sTM and other biomarkers like vWF and P-selectin in COVID-19 patients. 94 They also found that the level of sTM was higher than health controls and sTM level was correlated with disease severity. Overall, these studies demonstrated that endothelial damage is present in a wide range of COVID-19 patients, particularly as people become critically ill. Furthermore, sTM levels could be used a biomarker to identify which patients are most likely to progress toward critical illness and possibly death, as these patients might benefit from closer monitoring and possibly earlier intervention.

Pneumonia, community-acquired pneumonia: in the pathogenesis of community-acquired pneumonia (CAP), there is a high inflammatory response which causes damage to the endothelium leading to coagulation activation and the release of inflammatory mediators. 96 Common assessments of disease severity are the pneumonia severity index (PSI), used to identify low-risk patients or those who can continue to outpatient care, and the CURB65 score which is used to determined high-risk patients. 97 98 The measurement of related biomarkers has also been added to supplement the scores of these two assessments. Recently, plasma sTM levels have been shown to significantly increase in patients with worsening CAP. 99 Also, sTM levels used in combination with either the PSI or CURB65 score allowed for an increase in accuracy in prognosis evaluation. These results indicate that sTM level is useful in the evaluation of the severity and outcome of CAP in the emergency department.

Severe acute respiratory syndrome: SARS is a viral respiratory illness caused by a coronavirus called SARS-associated coronavirus (SARS-CoV). Liu et al evaluated classic plasma markers of endothelial injury tissue-type plasminogen activator (t-PA) and sTM in patients with SARS. 100 They found that sTM and tPA had significantly elevated levels in SARS patients in comparison to controls. Furthermore, patients who died had extremely high levels of sTM (1.01 nmol/L). Increased plasma concentrations sTM in patients with SARS suggest the possibility of endothelial injury. This observation is consistent with the new coronavirus disease COVID-19 which also shows higher sTM level in afflicted patients. 93 94 The sTM level may not only provide a useful treatment and prognostic index but also allow a further understanding of the pathological condition of the disease.

Sepsis: a key role of the pathogenesis of sepsis are uncontrolled inflammatory responses which causes damage to endothelial cells. 101 sTM levels have been used as diagnostic, prognostic, and mortality indicators in patients with sepsis. 102 103 Multiple studies have shown a positive correlation between sTM levels and the severity of sepsis in both adult and pediatric patients. 40 93 104 105 It has even been shown that sTM was better at predicting severe complications, such as multiple organ dysfunction syndrome (MODS), over accepted risk and prognosis assessment methods such as SOFA and APACHE II. 105 Additionally, patients who died of sepsis had higher levels of plasma sTM levels compared with those who did not. 40 106 From these studies, it can be inferred that sTM level can be used to track the severity of sepsis and possibly how the patient's disease will progress. Early diagnosis and treatment of people undergoing septic shock (SS) is crucial for their survival and can help reduce mortality rates. 96 MVs have been largely studied as potential biomarkers in SS. A recent case-control study found the trend of various MV subtypes during SS to evaluate their possible association with severity of illness and sepsis-related complications (DIC and acute kidney injury [AKI]). 72 Specifically, septic patients showed higher levels of all MVs considered compared with controls. TM + MV were significantly lower in more severe sepsis.

##### Cardiovascular Diseases

There is increasing experimental evidence that endothelial dysfunction represents an important component of cardiovascular disease (CVD) and stroke. In many cases, TM shedding from endothelial cells of arteries and veins contribute to certain amount of circulating sTM. Therefore, the levels of circulating sTM are highly related with CVD and stroke.

Abdominal aortic aneurysm: abdominal aortic aneurysm (AAA) is a vascular disease in which endothelial dysfunction plays an important role. 107 Brunelli et al reported a significantly higher level of sTM in AAA patients associated with elevated homocysteine levels, a factor alleged to contribute to endothelial injury. 86 Another study evaluated sTM concentration in patients undergoing a surgery for the repair of AAA and examined its association with disease severity reflected by aneurysm size. 108 It was found that sTM concentrations were significantly increased in AAA patients compared with healthy volunteers. This study demonstrated a significant increase in concentration of sTM in the blood of AAA patients which is in line with previous findings. 81 In particular, sTM concentration remained elevated in the subgroup of patients without clinical manifestations of atherosclerosis, suggesting that an increased sTM level is an independent feature of AAA rather than an effect of atherosclerotic alteration which commonly occurs among AAA patients.

Acute myocardial infarction: several studies have indicated an association between hemostatic markers and acute myocardial infarction. Öhlin et al reported that sTM antigen in plasma is increased in patients with acute myocardial infarction treated with thrombolytic therapy. 109 van Dreden et al studied plasma levels of 10 coagulation factors and analyzed the activity of plasma tissue factor (TFa), sTM, and procoagulant phospholipid in patients with acute myocardial infarction at the time of hospital admission. 110 It was found that plasma levels of TFa, sTM, and procoagulant phospholipid were significantly higher in cases of acute myocardial infarction than in healthy volunteers. In addition, patients with an unfavorable outcome during a 2-month follow-up had higher levels of TFa, sTM, and procoagulant phospholipid. The association of the level of the activity of these three factors may provide a useful tool to assess the prognosis of acute myocardial infarction.

Atherosclerosis: TM is expressed in a variety of cells associated with atherosclerotic lesions. These include endothelial cells, foamy macrophages, spindle cells, intimal smooth muscle cells, and medial smooth muscle cells. 18 An early study showed that serum levels of sTM were significantly increased in patients with an atherosclerotic lesion versus healthy controls. 100 A further significant increase was seen in patients who had multiple lesions. 100 It was also found that sTM positively correlated with vWF and was more sensitive to determining wide-spread disease than vWF. sTM levels could also be used as a predictor for developing atherosclerosis. A large cohort study found that patients whose plasma sTM levels were raised had a higher chance of developing carotid atherosclerosis. 111 It has also been shown that plasma sTM levels could be used as a biomarker to help determine if a patient with ischemic heart disease would develop a cardiovascular end point. 112 Patients who had hypercholesterolaemia, but no signs of atherosclerosis or other cardiovascular complications, were not seen to have any difference in levels of plasma sTM. 113 This indicates that sTM may not be a good predictor of atherosclerosis but may be useful for determining/monitoring disease severity.

Coronary heart disease: the relationship between plasma sTM and the relative risk of coronary heart disease (CHD) has been evaluated, and levels of sTM were seen to be inversely associated with the risk of CHD. 111 It was found that individuals with a high level of sTM were associated with a significant reduction in the relative risk of coronary heart disease events. Combinatorial analysis of sTM and soluble intercellular adhesion molecule-1 (sICAM-1), a known biomarker for CHD, provides a more specific assessment of CHD risk. In another large prospective case-cohort study, it was found that sTM did not predict future coronary events in apparently healthy, middle-aged patients. 114 Although not predictive, increased sTM concentrations on the incidence of coronary events among apparently healthy patients do not exclude the potential significance of sTM-regulated mechanisms in the pathophysiology of atherothrombotic heart disease.

Disseminated intravascular coagulation: biomarkers of endothelial damage have been previously seen to increase in DIC patients, including sTM, tissue type plasminogen activator (t-PA), and plasminogen activator inhibitor-1 (PAI-1). 115 Patients with DIC were shown to have nearly double the amount of plasma sTM levels compared with healthy controls. Plasma sTM levels were also significantly higher for patients whose condition worsened to develop organ failure or death. 41 101 116 117 sTM was better than t-PA, PAI-1, and vWF at correlating with the development of organ failure. 118 In addition, endothelial injury releases microparticle TM in the pathogenesis of DIC. 34 It was found that number of microparticle TM was increased significantly in both severe sepsis patients and controls. With an additional increase in International Society of Thrombosis and Hemostasis (ISTH) DIC score, the study suggests that the specific bioactivity of microparticle TM may play a role in the progression of sepsis-induced DIC. Therefore, sTM levels and microparticle TM can be used as biomarkers of DIC.

Thrombotic thrombocytopenic purpura (TTP)/hemolytic uremic syndrome (HUS): the plasma sTM levels were measured in patients with thrombotic thrombocytopenic purpura (TTP)/hemolytic uremic syndrome (HUS) and in healthy volunteers to examine the relationship between the occurrence of hemostatic abnormality or vascular endothelial cell injury and patient outcome. 119 It was found that the plasma sTM levels in TTP/HUS patients were significantly higher than in healthy volunteers. Furthermore, the plasma sTM levels were significantly higher in patients who died than in patients who survived. These findings suggest that the outcome of TTP/HUS is related to vascular endothelial cell injury and that plasma sTM levels may be useful markers for fatality of TTP/HUS patients who survived and those who died. On the other hand, increased plasma sTM levels were reported in HUS patients. 119 120 Since sTM is probably excreted via glomerular filtration, the impaired glomerular function present in HUS could contribute to the increased circulating sTM levels found in patients.

##### Diabetes Mellitus

With both endothelial damage and dysfunction at play in diabetes mellitus (DM), a significant amount of research has been performed on how sTM levels change in DM. The overall trend is that sTM levels are increased in the biological fluids of diabetic patients. 30 112 121 122 123 124 125 Levels were also shown to have a weak positive correlation of disease duration and number of complications. 112 122 123 However, no difference was found between type-I and -II diabetic patients. 122 It has also been shown that high sTM levels were associated with increased risk for all-cause mortality and CVD deaths. 126 Plasma and urinary sTM levels were also positively correlated with urinary albumin, a marker for nephropathy. 122 127 The subspecies profile of plasma sTM was also found to be different in diabetic patients compared with healthy person. In diabetic patients, more of the 74 and 48 kDa TM fragments were found, while five other fragments were found to be unchanged. 30 These different TM fragments formation indicates a complicated TM release mechanism, which deserves a further investigation.

##### Hypertension

Earlier studies on pulmonary hypertension found that the pulmonary vascular endothelium is deficient in anticoagulant proteins like TM. A later study with patients with chronic thromboembolic pulmonary hypertension (CTEPH) showed significantly lower sTM levels than that in the control group. 126 In contrast, there is no difference of the plasma sTM concentration of patients suffering from acute pulmonary thromboembolism (APTE). After patients underwent pulmonary thromboendarterectomy, the sTM concentration increased significantly. In the CTEPH group, the plasma sTM concentration was negatively correlated with pulmonary arterial pressure and total pulmonary resistance. 126 Another study confirmed that plasma sTM level was elevated in scleroderma associated pulmonary hypertension compared with scleroderma controls and healthy controls. 128 It is known that circulating levels of sTM are elevated in patients with hypertension in proportion to the severity of the vascular damage. 129 A cross-sectional study with patients with essential hypertension suggested that circulating levels of sTM were elevated in hypertensive patients as compared with normotensive subjects and that the sTM level may be a molecular marker of the latent progression of atherosclerosis in hypertensive patients. 130

##### Kidney Disease

The excretion of sTM from kidney will affect the concentration of the plasma sTM and urinary sTM. Therefore, kidney disease will affect the concentration of the plasma sTM and urinary sTM accordingly. In patients experiencing renal failure caused issues where endothelial cell damage does not occur, plasma sTM is raised. There is also a positive correlation with serum creatinine, a staple biomarker of renal function, and a negative correlation with creatinine clearance. 131 However, many kidney diseases do cause endothelial cell damage. Elevation of plasma sTM level and different sTM fragments have been confirmed in urine to related kidney diseases in several studies. 132 133 134 135 Chronic kidney disease (CKD) is linked with coagulation and inflammation dysregulation where TM is a key player. 136 137 For patients experiencing CKD, serum sTM levels were found to be positively correlated with disease severity after stage 3. The rise is thought to be due to increasing complications of CKD, such as atherosclerosis. In addition to the usual relation with serum creatinine, serum sTM levels were found to be negatively correlated with the estimated glomerular filtration rate. This may also be a cause for the increase in sTM levels. 133

##### Liver Diseases

The liver regulates the most chemical levels in the blood by breaking down or converting certain substances. There are considerations on liver function as predictors of sTM levels. Therefore, there is a correlation between sTM levels and liver diseases as well. Plasma sTM levels were often evaluated in patients with liver diseases. 138 139 140 141 TM expression in hepatic endothelial cells are highly affected in liver diseases like viral hepatitis 134 and liver damage 141 which also cause sTM release. Overall, liver enzymes could be modulators of sTM and sTM levels as well. The increase in plasma sTM levels in liver disease may be due to defective hepatic degradation of the circulating sTM. On the other hand, higher level or activity of liver enzymes may cause decreased plasma sTM levels. It is not known how liver function and dysfunction influence sTM levels. The plasma sTM levels and liver diseases deserve further mechanistic study. MVs have been proposed as potential biomarkers of cirrhosis. A recent study characterized circulating plasma MVs profile in patients with decompensated cirrhosis and AKI. 73 They found that patients with cirrhosis with AKI had a significantly higher level of total MVs compared with patients with cirrhosis without AKI but comparable severity of underlying liver disease. They concluded that AKI is responsible for the increased levels of MVs observed in patients with cirrhosis.

##### Lupus, Systemic Lupus Erythematosus

Common pathologic features that accompany systemic lupus erythematosus (SLE) are endothelial cell apoptosis, endothelial dysfunction, and inflammation. 142 143 144 A defining pathologic feature of SLE is widespread and recurring vascular lesions. 145 Markers for endothelial cell injury, like sTM, could be useful for helping determine the diagnosis or severity of the disease. 146 147 sTM levels as a whole are elevated in patients, both adults and juveniles, suffering from SLE. 95 148 TM levels also showed a positive correlation with disease activity and was stronger versus other markers such as E-selectin and sICAM-1. 146 148 149 150 151 Complications of SLE related with increasing sTM levels include nephritis, vasculitis, and central nervous system (CNS) lupus. 146 152 sTM levels were also useful in distinguishing between patients with active lupus nephritis (LN) or inactive LN. 153

##### Multiple Sclerosis

Determination of whether sTM levels change between healthy and multiple Sclerosis (MS) patients has had mixed results. Some research groups have seen no change in serum sTM levels or sTM levels in cerebral spinal fluid (CSF). 78 79 However, even though serum sTM levels and CSF fluid sTM levels alone were not significantly different among groups, a difference was seen in TM indexes. TM index takes into account the sTM levels in serum and CSF and is compared with albumin levels in the serum and CSF. TM indexes were higher in relapsing MS and progressive MS groups compared with healthy patients. It was attributed the increase in index as a result of endothelial cell damage or deregulation of TM release in the brain microvascular endothelial cells. 78 A few studies though have found significant changes in sTM levels in patients with MS and even change among differing disease states. 150 These results indicate sTM can be used to determine disease severity as sTM levels rise with more severe varieties of MS.

##### Obesity

Obesity is a complex disease and has high risk of other diseases and health problems, such as heart disease, diabetes, high blood pressure and certain cancers. The plasma concentration of sTM is associated with obesity as well. A previous study investigated the plasma concentration of sTM in children and adolescents with obesity. 154 They measured plasma concentration of sTM, blood lipids profile, creatinine, and its clearance. They found that plasma concentration of sTM in the group with obesity was significantly higher than that in the control group. There was no significant association between sTM and age or sex. In addition, statistically significant correlation between sTM and body mass index (BMI) was observed in the obese group.

##### Preeclampsia

TM is present on syncytiotrophoblasts and the endothelium of the vasculature that covers the trophoblastic surface. 155 156 TM is the main mediator of the anticoagulant system in the placenta. 157 Minakami et al first studied the plasma levels of sTM in preeclamptic women as compared with normal pregnant and nonpregnant women. 158 They found that the plasma levels of sTM were significantly elevated in preeclamptic women versus controls. Later studies also confirmed increased sTM levels of preeclampsia (PE) patients. 159 160 sTM levels also increase with each trimester in normal pregnancy which is made worse in PE complicated pregnancies. 161 162 Another observation was that plasma sTM levels began to significantly rise earlier in patients who would later develop PE by week 24 when compared with pregnancies that were uneventful by week 32. 163 The rise in sTM levels is thought to be mainly due to cleavage from the endothelial surface which is further supported by the finding that the placenta of PE patients expresses less TM on their endothelial surfaces versus normotensive patients. 164

##### Stroke

Plasma sTM levels and vWF were often measured to check the risk of ischemic and hemorrhagic stroke. 165 Earlier study found no relationship between increased sTM concentration and the risk of brain infarction (BI). 165 However, a later study confirmed that sTM level was associated with lacunar stroke and asymptomatic carotid stenosis progression. 166 Since then, several studies have confirmed increased plasma sTM levels in stroke. 43 167 A study of patients with acute cerebral infarction (ACI) in Japan confirmed that sTM concentrations were correlated with the severity of ACI. 167 It was found that sTM concentrations at admission in patients with cardioembolic infarction were significantly lower than those of lacunar infarction. Although sTM concentrations serve as a useful marker for endothelial cell damage, they are decreased in patients with severe ACI, especially in atherothrombotic and cardioembolic infarctions. Lower sTM concentrations may play some important role in disease progression or in the recurrence following ACI, although the exact mechanism of this unique result should be clarified.

##### Trauma-Induced Coagulopathy

The emergency management of acute severe bleeding in trauma patients has been paid more attention in recent years. In particular, a prompt assessment of coagulation alterations is necessary and allows for immediate hemostatic resuscitation procedures. 168 Coagulopathic bleeding stems from a complex interplay among hemostatic and inflammatory systems which are characterized by a multifactorial dysfunction in major traumas. Anticoagulation is one of the main determinants of trauma-induced coagulopathy (TIC). Brohi et al found increased levels of circulating TM, along with decreased plasma levels of protein C within 1 hour from a traumatic event in patients with severe anatomical injury and tissue hypoperfusion. 169 TIC remains one of the most diagnostically and therapeutically challenging conditions. Measurement of pathophysiological alterations in TIC will facilitate better emergency management of TIC.

##### Cancer

TM expression has been described in multiple cancer types on the endothelium and tumor cells. 170 171 It is known that TM exerts an influence on the metastatic capacity of cancer, with elevated TM expression conferring a positive predictive and prognostic factor. 170 The level of sTM has been evaluated for cancer metastasis and prognosis. 172 To clarify the correlation between sTM levels and clinicopathological parameters, the plasma sTM levels of primary soft tissue tumors (benign and soft tissue sarcoma [STS]) were measured before biopsy or treatment. It was found that STS tumors had significantly higher sTM concentration than benign tumors. These results demonstrated that a high level of sTM has the potential to be a significant predictor of metastasis and poor prognosis in STS patients. sTM is a candidate molecular marker for high metastatic potential and can be clinically useful for guiding therapeutic strategy which deserves future study. Portal vein thrombosis (PVT) is a common complication of hepatocellular carcinoma and is associated with a poor prognosis. Circulating MV-TM in plasma of patients with cirrhosis with and without HCC were evaluated for the possible contribution of MV-TM in PVT occurrence in HCC. 74 They found that patients with concomitant cirrhosis and HCC showed higher levels of MV-TM than patients with cirrhosis without HCC and healthy controls.

#### Circulating Thrombomodulin in Surgical Operation and Intervention

Surgical operation and intervention can cause endothelial damages and thus lead to sTM release from the cells and tissues of the injured sites. Therefore, sTM levels were often measured related to the disease treatment and recovery. This section summarizes the sTM levels related to variety of surgical operations and interventions. The sTM levels in surgical procedures are summarized in Table 4 .

**Table 4:** sTM in surgical procedures and transplantation

Surgical procedure/transplantation		Other markers	Reference(s)
Surgical procedure	Cardiac catheterization	Thrombin	211
	Coronary artery bypass graft (CABG)	IL6	173 174
	Percutaneous coronary interventions (PCI)	C-reactive protein (CRP)	175
	Bone marrow transplantation	sTM activity	53 55 84
Transplantation	Liver transplantation	Thrombin-antithrombin III complexes, protein C aminotransferase (AST), alanine aminotransferase (ALT)	177 178 179 184
	Renal transplantation	sVCAM-1, E-selectin, P-selectin, thrombomodulin, sICAM-1, sICAM-3, IL6, IL-8, TNF-α, CRP	182 212

Abbreviations: IL, interleukin; sTM, soluble thrombomodulin; sICAM, soluble intercellular adhesion molecule-1; TNF, tumor necrosis factor.

sTM levels were assessed during cardiac catheterization procedure. Vielhaber et al conducted a prospective study in children by measuring sTM concentrations, along with thrombin generation before, at the end of and 24 hours after cardiac catheterization. 170 They found that sTM concentrations increased significantly at the end of cardiac catheterization and returned to pretreatment levels 24 hours later. Data from this study indicate that increased sTM concentrations after cardiac catheterization are a sign of short-term endothelial damage. Endothelial damage caused by coronary artery bypass graft (CABG) procedure itself could contribute to bypass graft occlusion in the early postoperative period. A significant increase in sTM level during the first week post-CABG was observed. 173 This phenomenon might account for the increased risk of occlusion of bypass grafts at this moment of the postoperative period. Another recent study analyzed the association between levels of sTM and inflammation and described the possible explanations about association between sTM and postoperative complications. 174 They found that the levels of sTM increased during the first postsurgery week, and then decreased to levels similar to those recorded preoperatively. The transient increase in sTM during the first week after CABG was associated with an inflammatory response and leukocytosis. The levels of plasma sTM and inflammatory and myonecrotic markers in patients undergoing percutaneous coronary interventions (PCI) have been also evaluated. 175 Specifically, plasma levels of sTM, C-reactive protein (CRP), and creatine kinase and its MB isoenzyme were measured before and after PCI. As a result, sTM levels increased significantly after PCI, showing better correlation with inflammation than myocardial injury, indicating an endothelial origin.

#### Circulating Thrombomodulin in Transplantation

TM has been hypothesized to play a role in graft rejection as increase in TM expression has been shown to lower the risk of xenotransplantation failure 176 and graft rejection. 82 A 1995 study explored how plasma sTM levels changed with reperfusion and if sTM levels could predict graft complications. It was observed that plasma sTM levels after the anhepatic phase were triple those of preoperative levels. Additionally, a large incidence of graft rejection or failure was seen when sTM levels were greater than 138 ng/mL. 177 This section describes sTM levels related to different transplantations. The sTM levels in transplantation are summarized in Table 4 .

Liver transplantation can cause endothelial damage allowing sTM to serve as a marker during liver transplantation. 178 179 In addition, reperfusion injury causes damage to endothelial cells and leads to sTM release as well. Sido et al. evaluated intraoperative sTM as a marker of reperfusion injury in liver transplant recipients. 177 It was found that sTM levels were significantly elevated, as compared with healthy control patients, and remained unchanged at the end of the anhepatic phase. In addition, postreperfusion sTM levels correlated significantly with the early liver enzyme release (aspartate transaminase). These observations indicate that sTM is a marker of reperfusion injury which correlates with the early liver enzyme release and the accumulation of intrasinusoidal granulocytes. sTM level was used as marker for predicting early graft function in clinical liver transplantation. 180

Renal transplantation can cause endothelial damage and dysfunction that may contribute to the hypercoagulable and inflammation states presents in renal transplant. A recent study assessed sTM, vWF, and IL-6 in renal transplant recipients (RTRs) and associated their plasma levels with primary cause of end-stage renal disease (ESRD) and allograft function. 181 They found that sTM and IL-6 could be used as potential markers for evaluating renal graft function. sTM was more related to the primary cause of chronic kidney disease (CKD) compared with vWF and IL-6. In addition, sTM and other serum biomarkers of endothelial dysfunction and low-grade inflammation were evaluated for renal replacement therapy. 182

#### Circulating Thrombomodulin in Hemodialysis

There have been clinical studies suggesting a correlation between increased sTM levels and vascular endothelial damage in patients undergoing HD. Very high plasma levels of sTM were considered to be associated with endothelial cell damage in addition to the presence of uremia. 182 The HD procedure alone is suspected for release by and damage to the endothelial cells, probably of result of hypoxia, complement activation, platelet activation, and a release of leukocyte proteases during HD. Numerous coagulation and fibrinolytic disorders appear in HD patients. 181 It was found that coagulation factors TFPI, sTM, and vWF were increased in HD patients. 183 sTM levels can be used as marker for monitoring HD procedure.

### Summary and Future Perspective

TM is a type-1 transmembrane glycoprotein expressed mainly on vascular endothelial cells which plays many biological functions. TM also circulates as sTM and MV-TM in biological fluids ranging from serum and urine to synovial fluid. sTM can be generated by physical stress, enzymatic cleavage, or chemical cleavage of the intact protein, 28 50 while MV-TM is shed from cell membrane as membrane fragments containing the membrane TM. 35 In addition, TM mutations also cause TM release. 44 45 46 76 The circumstances in generating sTM are different between endothelial cells reacting to extracellular stimuli and a congenital genetic mutation in the *THBD* gene. In normal physiologic conditions, sTM circulates at a low concentration (<10 ng/mL) in plasma 31 39 ; however, it is elevated in several pathologic conditions associated with endothelial dysfunction. 34 66 On the other hand, MV-TM levels in serum have also been observed during systemic inflammatory response syndrome in humans and during heat stroke. 68 69 Therefore, increased plasma sTM levels and MV-TM have been used to monitor diseases development and surgical operation, transplantation, and even predict mortality in patients such as COVID-19.

It is still unclear how sTM levels contribute to physiology and pathophysiology. It will totally depend on what fragments of the sTMs are as each domain of TM has distinct activity which may be generated under specific conditions. However, it is unknown if the release of sTM is a tightly regulated process in which specific domains are cleaved and released. There have been four, 31 six, 184 or seven 30 sTM fragments reported in plasma, suggesting multiple cleavage sites and different mechanisms due to endothelial cell damage in varying diseases. In addition, two forms of sTM were isolated from human urine in two separate studies. 81 185 Concerning the biological activity, sTM isolated from plasma showed the thrombin-mediated activation of protein C and the activity was 30 to 50% compared with that of cellular TM. 186 Also, the sTM fragments in plasma inhibit fibrinolysis through the activation of TAFI. 187 Characterization of these fragments by *N* -terminal sequencing revealed that one form encompasses the EGF repeats and retained the ability to bind thrombin. In contrast, the second fragment corresponded to the equivalent molecular weight for the *N* -terminal CTLD and failed to bind thrombin. Overall, the precise functional relevance and activity of sTM fragments and the mechanisms for sTM release are not fully understood, mechanistic and proteomic study about sTM fragments merit further investigation. Finally, the physiological and pathological relevance of different sTM fragments require further investigation.

Different methods are used to quantify sTM concentration in biological samples. EIA and ELISA methods are the most common ways to measure sTM in which an anti-TM antibody is used. The main question is if a single antibody can capture all fragments of sTM when multiple forms of sTM exist in the biofluids. However, it is an unanswered question. In addition, TM activity, like protein-C activation, is also measured in biological samples for various diseases, which is often used in conjunction with sTM concentration measurements. Again, since different domains of TM have different activities and many fragments of sTM exist, a single activity assay may not be adequate to evaluate sTM in different diseases. On the other hand, MV-TM activity has not been investigated. It is known that MVs have different membrane assembly from cell membrane 188 which may affect TM activity on the MVs. Therefore, further research is needed to investigate circulating TM concentration and their activity for both basic research and clinical applications.

### Conclusion

Overall, sTM levels are widely investigated in disease monitoring and diagnosis. However, preexisting/coexisting conditions such as liver or kidney disease or both will affect plasma sTM levels after release from vascular endothelial injury. 189 The liver appears to be an important site of TM clearance as confirmed in experimental animals, 190 unfortunately, this process has not been adequately studied in humans. On the other hand, plasma sTM levels are also strongly dependent on renal excretory function. 132 135 It was reported that renal function serves as a determinant of plasma levels of endothelial markers. 191 It was found that there is a significant increase of sTM levels in conservatively treated renal patients without evidence of liver dysfunction. Therefore, interpretation of sTM levels in clinical studies should be performed with close attention to liver and renal function, and testing the markers of liver and renal functions is necessary for the clinical application. All these together illustrate the high demand for fully understanding of the precise structure domain, functional relevance, and activity of sTM and MV-TM in serum and other biofluids, all which will be very essential to comprehend their significance and role in diseases and can be explored as a biomarker for diagnosis and tracking diseases development, therapeutic efficacy and clinical outcome assessment.
