# Supplementary material for: Circulating Thrombomodulin: Release Mechanisms, Measurements, and Levels in Diseases and Medical Procedures
Source: TH Open. 2022 Jul 11;6(3):e194–212. doi: 10.1055/a-1801-2055 (PMC9273331; doi:10.1055/a-1801-2055)
Supplement: Supplementary file 1 — Supplementary Material [file 10-1055-a-1801-2055-s210080.pdf]

Supplementary Table S1. List of commercially available ELISA kit for measuring sTM

Name of kit	Detector antibody	Sample type(s)[a]	Sensitivity[b]	Company
Thrombomodulin (CD141) human ELISA kit (ab46508)	Biotinylated antithrombomodulin	Cell culture supernatant, serum, plasma	0.31 ng/mL	Abcam
Human thrombomodulin PicoKine ELISA kit	Biotinylated antihuman thrombomodulin	Cell culture supernatant, serum, plasma (heparin, EDTA, citrate) and urine	<10 pg/mL	Boster
Human thrombomodulin ELISA kit	Biotinylated antihuman thrombomodulin	NA	41.25 pg/mL	Biorbyt
TM (CD141) human ELISA Kit	Biotinylated antithrombomodulin	Cell culture supernatant, plasma, serum	< 0.38 ng/mL	BioVendor
Human TM ELISA kit	Biotinylated antihuman thrombomodulin	Serum, plasma, cell culture supernatants	< 0.31 ng/mL	Cell Sciences
Human thrombomodulin/BDCA-3 DuoSet, 15 plate	Biotinylated mouse antihuman thrombomodulin antibody	Cell culture supernatant, serum, and plasma	50 ng/mL	R&D Systems
Human thrombomodulin ELISA kit (ab214029)	HRP-labeled antibody (thrombomodulin conjugate)	Cell culture extracts, cell culture supernatant, Cit plasma, EDTA plasma, hep plasma, serum, tissue extracts, urine	3 pg/mL	Abcam
Human thrombomodulin/BDCA-3 Quantikine ELISA kit	HRP-labeled antibody (thrombomodulin conjugate)	Serum, heparin plasma, cell culture supernates, cell lysates, citrate plasma, EDTA plasma, urine	27 pg/mL	R&D Systems
Human thrombomodulin ELISA kit	Biotinylated Detection Antibody	Serum, plasma and other Biological fluids	37.5 pg/mL	Abbexa
Human thrombomodulin matched antibody pair kit (ab219526)	Biotinylated detection antibody	NA	9.96 pg/mL	Abcam
THBD (human) ELISA kit	Biotinylated detection antibody	Cell culture supernatant, plasma, serum	<0.31 ng/mL	Abnova
Thrombomodulin ELISA kit (human) (OKAG00252)	Biotinylated detection antibody	Cell lysates, plasma, serum	32 pg/mL	Aviva
Human thrombomodulin ELISA kit	Biotinylated detection antibody	Plasma	18.24 pg/mL	Biomatik
Human CD141/thrombomodulin ELISA set	Biotinylated detection antibody	Cell supernatants, buffered solutions, serum, plasma and other bodily fluids	<0.31 ng/mL	Cell Sciences

 © 2022. The Author(s).

Supplementary Table S1. (*Continued*)

Name of kit	Detector antibody	Sample type(s)[a]	Sensitivity[b]	Company
Human thrombomodulin ELISA kit	Biotinylated detection antibody	Serum, plasma, tissue homogenates	0.078 ng/mL	CUSABIO Technology
Human sCD141 ELISA kit	Biotinylated detection antibody	Serum, plasma, cell culture	0.31 ng/mL	Eagle Bioscience
Human TM ELISA kit	Biotinylated detection antibody	Serum, plasma, cell culture supernatant, tissue, other fluid	37.5 pg/mL	Elabscience
Human thrombomodulin ELISA kit (GWB-SKR252)	Biotinylated detection antibody	Cell lysates, serum and plasma	32 pg/mL	GenWay Biotech
Human THBD/CD141/thrombomodulin ELISA kit	Biotinylated detection antibody	Cell culture supernatants, citrate plasma, EDTA plasma, heparin plasma, serum, urine	10 pg/mL	Life Span BioSciences
Human TM (thrombomodulin) ELISA kit	Biotinylated detection antibody	NA	37.5 pg/mL	Nordic BioSite
Human thrombomodulin/BDCA-3 ELISA kit	Biotinylated detection antibody	Serum, plasma, body fluids, tissue lysate or cell culture supernatant	5.0 pg/mL	Novteinbio
Human thrombomodulin ELISA kit	Biotinylated detection antibody	Cell culture supernatant, serum, plasma(heparin, EDTA, citrate) and urine	<10 pg/mL	OriGene Technologies

Abbreviations: ELISA, enzyme-linked immunosorbent assays; NA, not available; sTM, soluble thrombomodulin.
Note: These ELISA kits use biotin- and HRP-labeled anti-TM antibodies or biotin-labeled detection antibodies to detect the sTM in serum, plasma, cell culture supernatant, tissue or other fluids. However, there is no information available related to the antibody specificity to the TM domains for all these ELISA kits.
[a,b]All from the vender's web site.

 © 2022. The Author(s).
